# mUbiSiDa: A Comprehensive Database for Protein Ubiquitination Sites in Mammals

**DOI:** 10.1371/journal.pone.0085744

**Published:** 2014-01-17

**Authors:** Tong Chen, Tao Zhou, Bing He, Haiyan Yu, Xuejiang Guo, Xiaofeng Song, Jiahao Sha

**Affiliations:** 1 Department of Biomedical Engineering, Nanjing University of Aeronautics and Astronautics, Nanjing, China; 2 State Key Laboratory of Reproductive Medicine, Department of Histology and Embryology, Nanjing Medical University, Nanjing, China; CSIR-Institute of Microbial Technology, India

## Abstract

**Motivation:**

Protein ubiquitination is one of the important post-translational modifications by attaching ubiquitin to specific lysine (K) residues in target proteins, and plays important regulatory roles in many cell processes. Recent studies indicated that abnormal protein ubiquitination have been implicated in many diseases by degradation of many key regulatory proteins including tumor suppressor, oncoprotein, and cell cycle regulator. The detailed information of protein ubiquitination sites is useful for scientists to investigate the mechanism of many cell activities and related diseases.

**Results:**

In this study we established mUbiSida for mammalian Ubiquitination Site Database, which provides a scientific community with a comprehensive, freely and high-quality accessible resource of mammalian protein ubiquitination sites. In mUbiSida, we deposited about 35,494 experimentally validated ubiquitinated proteins with 110,976 ubiquitination sites from five species. The mUbiSiDa can also provide blast function to predict novel protein ubiquitination sites in other species by blast the query sequence in the deposit sequences in mUbiSiDa. The mUbiSiDa was designed to be a widely used tool for biologists and biomedical researchers with a user-friendly interface, and facilitate the further research of protein ubiquitination, biological networks and functional proteomics. The mUbiSiDa database is freely available at http://reprod.njmu.edu.cn/mUbiSiDa.

## Introduction

Protein ubiquitination, known as the important protein post-translational modification of targeting proteins by ubiquitins for their subsequent degradation in the ATP-dependent ubiquitin proteasome system (UPS), plays an important role in cell activity [1]–[5]. Currently, many regulatory functions of protein ubiquitination have been discovered, including the regulation of DNA repair and transcription, control of signal transduction, and implication of endocytosis and sorting. There are also sufficient evidences showing that the deregulation of protein ubiquitination have been implicated in many diseases by degradation of many key regulatory proteins, including tumor suppressor, oncoprotein, and cell cycle regulator [6]–[8]. Therefore protein ubiquitination has captured the attention of researchers in life science fields.

Protein ubiquitination is implemented by ubiquitin binding to the lysine site of a target protein. The location, numbers, and distribution of ubiquitination site are important information for scientists to investigate the mechanism of UPS and relevant diseases [9], [10]. Therefore recently scientists began to focus on collecting and managing the available protein ubiquitination information. The current available ubiquitination database like UbiProt provides information almost exclusively on yeast protein ubiquitination due to the previous experimental limits [11]. For human, hUbiquitome mainly focused on the relationship between ubiquination enzymes and substrates, and only 279 substrates were included in the database [12]. With the recent new technology advances, such as proteomic technology, mass spectrometry technology, the growing number of the experimentally confirmed ubiquitination sites of mammalian protein laid the solid basis for investigating the regulatory mechanism of protein stability. Therefore it is imperative for scientific community to construct the comprehensive database for depositing and retrieving mammalian protein ubiquitination sites.

Consequently, in this study we constructed a user-friendly database, mUbiSida, which meets the above requirements. The dataset in mUbiSiDa are mainly collected from published papers. In total, we searched and obtained 104 references containing experimentally validated 35,494 mammalian ubiquitinated proteins from 5 species. Over 95% of these sites are from human and mouse. This comprehensive database enables not only the information retrieval of protein ubiquitination sites, but also the study of cross-regulation between post-translational modification [13], [14]. In addition, the mUbiSiDa can facilitate biologists in further exploration on the molecular mechanisms of protein stability related cell process.

In conclusion, the aim of the mUbiSiDa database is to provide a user-friendly web interface to browse, search, retrieve and update information on mammalian protein ubiquitinated sites, and to promote the further research of protein ubiquitination, biological networks and functional proteomics.

## Materials and Methods

### Data sources and database construction methods

**Figure 1:**
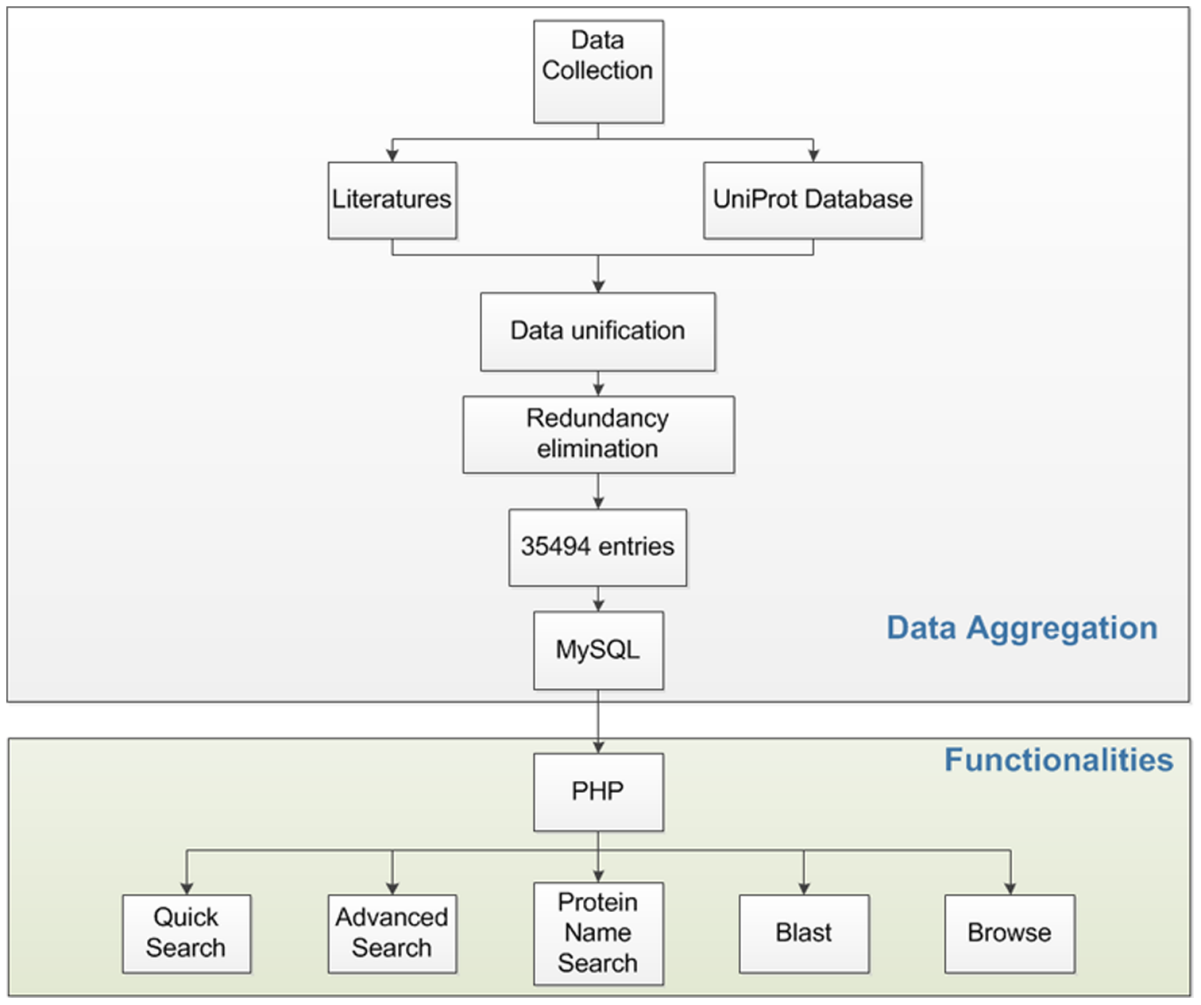
Data collection and the structure of mUbiSiDa.

**Figure 2:**
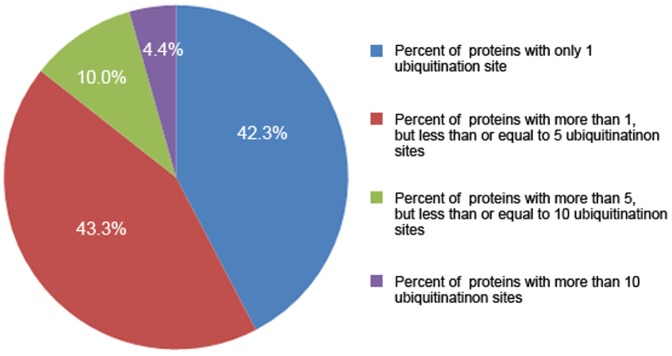
Statistics of protein ubiquitination sites.

Our data are collected from two main resources: Firstly, published literatures from PubMed. Through keyword search results in PubMed, we have obtained a mass of related literatures, after which experimentally identified mammalian ubiquitylation sites were downloaded from these literatures Several main references were listed in the section of References [15]–[26]. A well-known international database UniProt was also searched for protein ubiquitination [11]. Next, we manually reviewed entries of UniProt, and added those ubiquitinated proteins with reference support to the database, and eliminated those predicted lysine modifications without references support in Uniprot. Based on above data collection, we constructed a database containing 35,494 proteins from 5 species with 110, 976 ubiquitination sites. And currently there are 30,322, 5,168, 2, 1 and 1 counts for Human, Mouse, Bovine, Rat and Pig, respectively. The data collecting pipeline and the structure of mUbiSiDa were shown in Figure 1. The distribution of all experimentally validated mammalian protein ubiquitination sites collected in mUbiSiDa is shown in Figure 2. Proteins with less than or equal to 5 sites occupy 85.6% of the entries in mUbiSiDa. Proteins with greater than 5 and not greater than 10 sites occupy 10.0% of the entries. And proteins with greater than 10 sites occupy only 4.4%.

mUbiSiDa was constructed and configured upon a typical LAMP (Linux + Apache + MySQL + PHP) platform. Apache5.0.51b was firstly used to build up a webserver. All dataset were stored in MySQL 5.0, and web interface was achieved by PHP scripts (PHP version 5.2) on Linux, powered by an Apache server. WebPages were designed with html and JavaSript techniques. Website and database were connected and all kinds of function were achieved by PHP techniques.

## Results

### Functions and utility

**Figure 3:**
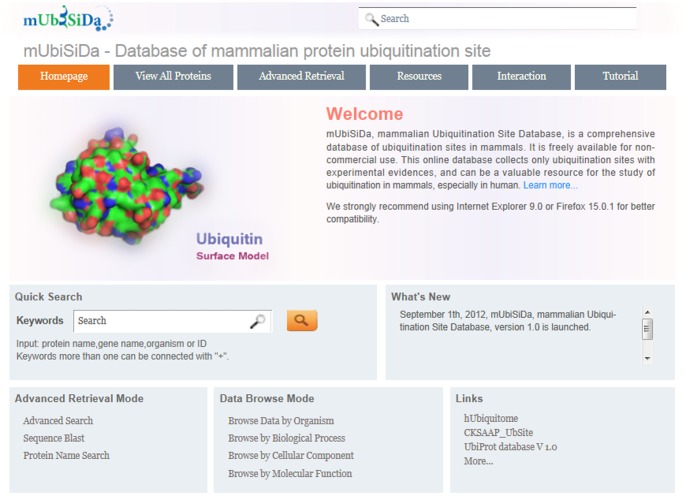
Home page of mUbiSiDa.

All functions of mUbiSiDa were shown in Home page (Figure 3). As an interactive, comprehensive and user-friendly database, mUbiSiDa mainly provides the following functions for users: Search, Advanced Retrieval, Browse, Resource, Interaction and Blast Search.

### Search function

In the text field of the Search page, the users can input the query strings, such as protein ID, protein name, or others, and then the obtained result pages will be the list of protein entries that matched the query strings. Those keywords matching the query strings in the result pages will be yellow highlighted. The users can view the detailed information about their interested protein by clicking the protein ID on the left column. For further retrieval, combined search is also available, but the most two query strings combined by ‘+’ are only supported in the current version homepage of database. In addition, on each result page, mUbiSiDa also provides customized function to reorganize the feature list of protein entries according to user's interests.

### Advanced retrieval

There are three options in Advanced Retrieval: (1) Advanced Search (2) Protein Name Search, (3) Sequence Blast, which is efficient access for the users to obtain their interested information. The users can obtain the specific protein ubiquitination information by entering more restricted words in six text fields combined with ‘AND’, ‘OR’ and ‘BUT’ in “Advanced Search” query page. We specifically designed “Protein Name Search” query page for the users conveniently to obtain their interested protein when knowing their names. There are four text fields for users to input query strings, part or all of the text fields can be used for combined search. When the ID text field is empty, the other three text fields were designed to be fuzzy query. When the ID text field is not empty, it was set to be precise search. The ID is supposed to be UniProt ID.

“Blast search” was also specifically designed for the users to predict the potential ubiquitination site information of new protein in other mammalian species, which is orthologs or paralogs deposited in the database. As a tool for comparing primary biological sequence information, Sequence Blast enables a user to compare a query sequence with all sequences of mUbiSiDa, and identify sequences of mUbiSiDa that resemble the query sequence above a certain threshold. By means of this method, one or few results which sequence is most similar to the input sequence will be obtained after blast process. The Sequence Blast is a high efficient tool for retrieving possible ubiquitinated lysine sites of proteins input by users. Not only will the protein ID, but also related references are posted after using blast. Users can view detailed information of the result by clicking the proteins ID. This tool extends the function of the mUbiSiDa to be used to predict protein ubiquitination sites for most mammalian species.

### Data browse and download

The dataset in mUbiSiDa can be browsed in four options: (1) Browse data by organism, (2) Browse data by biological Process, (3) Browse data by cellular component, (4) Browse data by molecular function.

In “Browse Data by Organism” page, the total dataset in mUbiSiDa are grouped by organism, which is listed in a table with 5 kinds of organisms collected by mUbiSiDa. The corresponding entry number of each organism is also listed. The users can view all associated entries for one organism by clicking on right numbers. This function is quite useful for users to view all the relevant information in particular species.

The total dataset in mUbiSiDa can also be viewed by Gene Ontology (GO). In the result pages, the gene ontology IDs classified as biological process, cellular component, and molecular function for each protein deposited in mUbiSiDa will be listed. The gene ontology terms, and the corresponding protein entries numbers are also listed. The user can select and click the gene ontology ID to view all proteins of the selected gene ontology ID. The matched results can help the users with a convenient way to view ubiquitinated proteins grouped by GO terms. The user can benefit from this function to do cell research on regulation of protein's function or biological processes.

All the dataset in mUbiSiDa can be downloaded at “Resources” web page.

### Submission of new protein ubiquitination site

In order to maintain an up-to-date and comprehensive resource, we designed the submission page for the users to submit their own data to mUbiSiDa. The submission page requires that the users should offer the following submission items: protein name, ID, organism, ubiquitination site(s), PubMed ID, GO ID, sequence, and other information.

### Detailed information in each entry page

**Figure 4:**
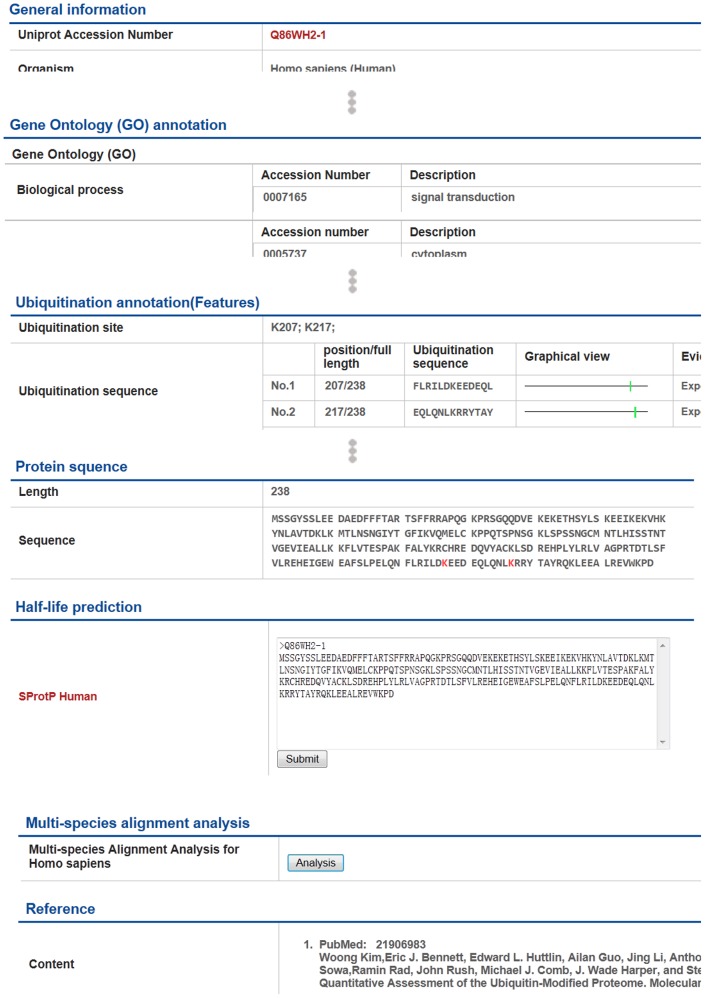
Detailed information page.

Users are allowed to have an access to the detailed information of each entry by clicking the ID on the left column in the result page. All information was carefully checked manually to ensure the accuracy of each entry. The each entry page is divided into six sections. The first section (Figure 4) provide basic protein information including “Uniprot Accession Number”, “Organism”, “Protein names” and “Gene names”. The Ontology section mainly contains the Gene Ontology (GO) of each entry, which was chiefly downloaded manually from UniProt. The most important part of mUbiSiDa is shown in Ubiquitination annotation (Features) section (Figure 4) including the location in the sequence about ubiquitination sites. In this section, all ubiquitination sites of lysine in the sequence are listed in the Graphical view. On the right of each ubiquitination sites, colored PubMed ID with hyperlink to Reference section is also provided as the strong evidence of protein ubiquitination site. All lysine ubiquitination sites were labeled and red colored in “Protein sequence” section. This makes it easy for users to recognize all ubiquitination sites. Besides, “Half-life Prediction” section and “Multi-species Alignment Analysis” section were added here for study of short-lived proteins and homologous sites in non-human species.

## Discussion and Conclusions

mUbiSiDa, the comprehensive mammalian ubiquitination site database, aiming to systematically aggregate all experimentally verified ubiquitinated protein, and provide biologists for analyzing protein stability and mechanism of protein degradation. Most data in this database are collected from human and mouse. It needs emphasizing that potential ubiquitinated lysine site from other organisms can be predicted according to the results of BLAST SEARCH or Multi-species Alignment Analysis. As a tool designed to be used by biologists and researchers, mUbiSiDa will be continually improved and updated to ensure the convenience and utility of the service, accuracy of the information and innovative in style and function.

To obtain an overview of the biological features of human and mouse ubiquitinated proteins, we firstly classified these proteins according to Gene Ontology (See File S1). For human, most proteins are located on intracellular part (61.9%). And the largest parts for molecular function and biological process were binding (33.9%) and metabolic process (27.2%) respectively. The distribution of mouse ubiquitinated proteins was similar to human, suggesting that the biological functions of ubiquitinated proteins may be conserved among mammals. We further performed functional enrichment analysis based on human and mouse ubiquitinated proteins using WebGestalt [27]. Among the top ten enriched pathways for human proteins, the most significant pathway is “Genes involved in Gene Expression”, which is also enriched among mouse proteins. Thus, we showed that mUbiSiDa could provide resources for further analysis of the functions of ubiquitination.

In the future work, efficiency in search process and new style of search need to be improved to satisfy different needs. The function of mammalian ubiquitination sites prediction will be subsequently added to mUbiSiDa in the future version. With continuous improvement, mUbiSiDa is expected to make a contribution to the researches on regulation of protein's function or biological processes.

## Supporting Information

File S1 **Gene Ontology classification and pathway enrichment analysis results for human and mouse ubiquitinated proteins.**
(XLSX)
